# Supplementary material for: Are carbon emissions trading and green financial instruments synergistic? -Comprehensive quantitative research based on content analysis
Source: PLoS One. 2024 Mar 7;19(3):e0298601. doi: 10.1371/journal.pone.0298601 (PMC10919601; doi:10.1371/journal.pone.0298601)
Supplement: S1 Data — (ZIP) [file pone.0298601.s001.zip › Data/地级市Z_F工作报告.zip!/╕ú╜¿/─╧╞╜╩╨/└┤╫╘╣1⁄2╓┌║┼í╛┬φ┐╦╩2╛▌═°í┐_2017.docx]

各位代表：

现在，我代表南平市人民政府，向大会报告政府工作，请予审议，并请市政协各位委员和其他列席人员提出意见。

一、过去五年和2016年的工作回顾

五年来，在省委、省政府和市委的正确领导下，我市各级政府认真学习贯彻党的十八大和十八届三中、四中、五中、六中全会精神，学习贯彻习近平总书记系列重要讲话精神和对福建工作的重要指示，主动适应经济发展新常态，抢抓机遇、拼搏进取，基本完成了市四届人大各次会议确定的目标任务。

——绿色发展富有成效。坚定不移地实施绿色发展战略，努力走出一条经济发展与生态环境保护相协调的道路。全市生产总值、规模以上工业增加值、一般公共预算总收入、地方一般公共预算收入年均分别增长9.5%、11.6%、9%、11.8%，全社会固定资产投资实现翻一番。产业结构进一步优化，加快培育现代农业，加快发展“5+3”等二、三次产业，净增产值亿元以上工业企业315家，新增龙泰竹业、华闽南配、闽瑞环保等“新三板”挂牌企业11家，旅游接待总人数、旅游总收入均实现翻一番。创建国家级生态县4个、省级生态县5个，森林覆盖率从71.1%提升至77.3%，森林蓄积量从1.34亿立方米增加至1.65亿立方米，先后被确定为节能减排等六个国家级示范试点城市，生态环境质量保持全国全省前列。

——基础设施日臻完善。合福高铁顺利通车，南三龙、衢宁铁路正在抓紧建设，建成、在建和列入国家中长期路网规划的铁路总里程突破一千公里；龙浦、宁武、松建、京台、邵光、延顺等6条高速相继建成通车，南平联络线、顺邵高速正在加快建设，建成、在建高速公路总里程达一千多公里，铁路、高速公路通车里程居全省首位，国道、省道和农村公路提升工程加快推进，实现了县县通高速、镇镇通干线、村村通客车；武夷山机场改扩建工程竣工，武夷山新机场选址正式获批。闽江航道（南平段）整治工程列入国家“十三五”发展规划。一批防洪排涝、水库除险加固和中小流域治理工程相继建成，供水、供气设施不断改善，城乡电力保障能力全面提升。

——城乡发展更加协调。把握国务院批准南平市行政区划调整的重大机遇，统筹加快武夷新区、延平城区和县域中心城市、美丽乡村建设。按照“绿色、特色、现代”三大建设理念，坚持“先谋后动、经营运作、集中集聚”三大原则，突破“征迁、资金、人才”三大难题，以科学的态度、磅礴的气势、优良的质量、高效的进度，全面推进武夷新区的开发建设，先后筹集各类资金295亿元，基本完成童游、将口、兴田三大组团基础设施和市政设施建设，南林核心区和武夷高新技术园区一批城建项目和产业项目相继建成或落地建设，武夷新区中心城市的框架初步形成。按照“统筹规划、集中集聚，先急后缓、加快实施，社会参与、公司运作，自我平衡、封闭运行”的原则，全力推进延平城区建设，突出老城区做“减法”，完善城市功能，补齐发展短板；新城区做“加法”，加快市政基础设施和产业园区建设，延平城区产城融合发展的条件已初步形成。各县（市、区）按照“六个一”要求，加快中心城市市政基础设施和产城一体化建设，立足生态环境条件，实施“百千万”工程，建设美丽乡村，城乡面貌明显改善。

——改革开放深入推进。新一轮市县政府机构改革顺利完成，事业单位分类改革有序推进。行政审批制度改革持续深化，率先在全省设区市公布“三张清单”，项目审批提速提效。按照“优化资源配置，强化资产运营”的原则，整合资源、突出主业、做大规模，组建8家国有集团公司，企业总资产780亿元、净资产376亿元。医药卫生体制改革持续深化，率先在全省启动公立医院药品耗材零差率改革。财税体制改革稳步推进，“营改增”试点全面推开。全面开展不动产登记，实现房、地、林发证全覆盖。发挥“万里茶道”双起点和武夷山陆地港优势，主动融入福建自贸区和“海丝”核心区建设，全方位、多层次的对外交流格局初步形成。

——社会保持安定稳定。依法治市进程加快，“六五”普法全面完成，荣膺“全国法制宣传教育先进城市”称号。社会治理创新取得成效，医患纠纷“南平解法”在全国推广，“民生110”网格化服务机制、农村社会治理“1+3”工作机制不断提升，“四无”平安村创建工作在全省推广。“平安南平”建设持续深化，社会治安综合治理考评连续三年位居全省第一。双拥工作走在全省前列，连续四届蝉联全国双拥模范城光荣称号。

——民生保障不断增强。教育“双高普九”提前实现，累计新建中小学校19所。率先在全省建立医疗救助“一站式”服务平台，累计改扩建县级医院24家。养老、医疗、生育等城乡一体化社会保障体系基本建立，保障性安居工程全面完成省下达任务，兜底保障政策不断完善。累计新增城镇就业12.37万人、转移农村劳动力27.62万人。坚持把脱贫攻坚作为第一民生工程，按照“六个精准”、“五个一批”的要求，扎实推进脱贫攻坚，累计脱贫18.46万人、造福工程搬迁12.05万人。民生支出在一般公共预算支出中的占比超过75%，累计完成为民办实事项目133项。城镇居民、农村居民人均可支配收入年均分别增长9.3%和10.9%。

2016年是本届政府任期届满之年，全市各级政府认真贯彻省委、省政府和市委的决策部署，采取有力措施，全力以赴应对经济下行压力。初步预计，全市生产总值1430亿元、增长7%左右；一般公共预算总收入119.26亿元、下降4.03%，地方一般公共预算收入82.97亿元、下降4%；固定资产投资1700亿元、下降4.3%；外贸出口73.9亿元、增长6%，实际利用外资1.63亿美元、增长12.2%；社会消费品零售总额557亿元、增长10.5%；城镇居民、农村居民人均可支配收入28000元、13270元，分别增长7.2%、8.2%。

一年来的主要工作和成效是：

（一）重点项目有力推进。坚持抓项目补短板扩投资，成立项目建设征地拆迁、重点项目前期工作、工程建设协调管理、筹融资工作、补短板工程包等领导小组，组建PPP管理中心，建立重点项目建设工作推进微信群，加快项目前期，实施征迁攻坚，拓宽筹融资渠道，推动项目建设提速增效。列入省市“五个一批”项目2058个、总投资7589亿元；在建行动计划重大项目176个，完成投资307亿元；省市重点项目346个，新开工98个，建成或部分建成94个，完成投资511亿元；24个工程包完成投资106亿元。

（二）城乡建设步伐加快。南平“一市两区”总体规划编制完成。武夷新区行政中心搬迁所必需的39个总投资293亿元的重大项目加快推进，高铁武夷山东站、客运枢纽、大武夷旅游集散中心、航天体验馆、崇阳溪景观带示范段、赤岸南林安置房、中小学幼儿园四所学校、武夷高新技术园区互联网经济产业园等一批项目基本建成，303快速通道、绕城高速、轨道交通、万达城市综合体、云谷小区、文化艺术中心、水资源配置和供电工程等项目正在加快建设。

延平城区规划设计全面优化提升，着手规划设计实施老城区“两横四纵”主街道立面改造和一批隧道、桥梁、公共停车场、农超市场、城市道路等补短板项目；着手规划设计实施新城区“一湖三江六岸”景观整治和九峰山、马头山森林公园项目以及高铁北站片区、延平新城港区、南平工业园区建设项目；启动实施连接新老城区的316国道城市快速通道改造项目。

各县（市、区）中心城市积极开展旧城改造和新城建设，结合中小流域治理和城乡污水、垃圾处理等环保设施建设，以及高铁、高速公路、江河沿线的环境整治，策划实施城乡建设补短板项目425个、总投资131亿元。新一轮“千村整治、百村示范”美丽乡村建设工程加快推进，“万人保洁”机制进一步完善提升。拆违治违力度加大，全市拆除“两违”面积333.5万平方米。

（三）产业发展提质增效。实施创新驱动发展战略，推进供给侧结构性改革，加快培育新兴产业，推动传统产业转型升级，实施电子信息、两化融合、信息消费等项目55项，实施技改重点项目75项。海川药业在韩国上市，新增“新三板”挂牌企业5家。推动企业技术、管理和营销创新，加快打造创新平台，建立2个国家级企业技术中心，元力活性炭、三金电子等37家企业入选省“科技小巨人领军企业”。注册地理标志商标4件、认定省著名商标25件。加快以旅游为主的服务业发展，新增国家3A级以上旅游景区7家，旅游接待总人数3408万人、增长16.5%，旅游总收入446亿元、增长20%。

（四）招商引资力度加大。围绕增强内生动力，持续加大招商引资力度，组织赴北京、深圳、澳门等地，开展以商引商、产业链招商、平台招商和小分队招商，积极参加“6·18”项目成果交易会、“9·8”投洽会，成功举办“11·16”茶博会，引进武夷新区万达文旅综合体、中国人寿国寿嘉园、大容量动力锂电池、邵武肉牛良种繁育加工等重大项目，全年引进投资亿元以上签约项目203个、总投资719亿元。坚持招商引资与项目落地并重，投资2000万元以上招商项目新开工274个。

（五）农业农村稳步发展。加快推动传统农业向现代农业转型，加快建设各县（市、区）现代农业科技园、农业示范区、农民创业园和农民创业示范基地，农业产业化、规模化程度进一步提升。加快培育“休闲农业”“定制农业”等新型农业业态，新增省级以上农业产业化重点龙头企业56家，邵武农业科技园获批为国家级农业科技园区，建瓯获评国家农村产业融合发展试点示范县。农村水利设施、路网工程和电网改造工程加快实施，农村生产生活条件进一步改善。

（六）脱贫攻坚成效明显。坚持精准识别、精准施策、精准管理，建立和完善“领导牵头、部门参与、上下一体、互动联动”的工作机制，用好用足中央、省扶贫开发政策，加快造血式、减负式、兜底式和搬迁式扶贫，统筹整合使用各部门资金，让有限的扶贫资源发挥出最大的效益。5个省级扶贫开发重点县争取帮扶资金12.86亿元，完成3.1万人脱贫任务和1.73万人“造福工程”扶贫搬迁任务。

（七）生态环境持续优化。武夷山国家公园体制、生态系统价值核算，武夷山、顺昌、建阳重点生态区位商品林赎买，光泽县党政领导干部自然资源资产离任审计等国家生态文明试验区试点工作扎实开展。环境保护目标责任制全面落实，水、大气和土壤污染防治行动计划有效实施，城乡黑臭水体、生活污水和畜禽养殖污染整治力度加大，主要流域水质功能优良；淘汰黄标车6347辆、燃煤锅炉164蒸吨，超额完成省下达任务，空气质量优良；土壤污染修复与治理试点工作扎实开展，水土流失综合治理39.16万亩，茶山整治有序推进，土壤环境质量安全得到保障。21个国家节能减排示范项目加快建设。科学划定生态红线，林业自然保护区占国土面积比例达7.04%以上，完成造林绿化22.2万亩，林业碳汇交易在全省率先突破。

（八）民生福祉不断增进。医疗保障管理体制改革扎实推进，全民参保登记试点工作基本完成。提前一年全面通过“全国义务教育发展基本均衡县”认定，中小学校扩容改造新增学位6115个，新增公办幼儿园15所。公共文化服务体系不断完善，朱子文化“一区五工程”保护与建设取得实质性进展。保障性安居工程新开工8118套、基本建成8995套，均超额完成省下达任务。第三届市运会、武夷山国际马拉松等赛事成功举办，群众体育和竞技体育协调发展。21项为民办实事项目全面完成。新增城镇就业2万人，农村劳动力转移就业4.7万人。安全生产形势稳定向好，食品药品安全有效保障，开展“平安南平”系列专项行动，刑事发案大幅下降，人民群众安全感明显提升。

（九）政府建设得到加强。深入开展“两学一做”学习教育，牢固树立“四种意识”，积极践行“三严三实”，认真落实中央八项规定精神，“三公”经费有效控制。党政机关公务用车制度改革全面推行。深化机关效能建设和行政审批制度改革，持续推进“放管服”改革，推行“双随机一公开”机制，强化事中事后监管，政府公信力和执行力显著提升。自觉接受人大监督、政协监督和社会监督，办理人大代表议案2件、建议124件，政协委员提案264件，办复率均为100%。坚持依法治市，深入推进依法行政，加大行政监察、审计监督和政务公开力度，扎实推进党风廉政建设和反腐败工作。

各位代表，五年的成绩来之不易。这是省委、省政府和市委正确领导的结果，是全市人民齐心协力、奋力拼搏的结果。在此，我代表市人民政府，向全市人民，向各民主党派、各人民团体和社会各界人士，向中央和国家机关、省直驻延机构，向人民解放军和武警驻延部队，向关心支持南平发展的港澳同胞、台湾同胞、海外侨胞和国际友人，表示崇高的敬意和衷心的感谢！

在充分肯定成绩的同时，我们也要清醒地认识到发展中还存在着不少困难和问题。主要是：发展的内生动力、动能不足，经济总量不大、发展速度不快的问题仍然十分突出，缺少产业大项目好项目；城乡规划建设管理水平不高，基本公共服务短板问题比较突出；社会保障和治理水平不高，教育卫生事业发展满足不了人民群众的需求；服务经济社会发展高端人才十分缺乏，营商环境不尽人意。这些问题的产生，主要是政府职能转变和体制机制创新不到位，一些工作人员素质、能力、作风适应不了当前发展形势的需要，服务改革创新和发展的意识不够强，慢作为、不作为甚至是乱作为的问题时有发生。对此，我们必须高度重视，采取有效措施认真加以解决。

二、今后五年和2017年工作目标任务

今后五年，是我市全面建成小康社会的决胜期。政府工作的指导思想是：深入学习贯彻以习近平同志为核心的党中央治国理政新理念新思想新战略，全面贯彻党的十八大和十八届三中、四中、五中、六中全会精神，统筹推进“五位一体”总体布局和协调推进“四个全面”战略布局，坚持稳中求进工作总基调，全面落实新的发展理念，按照省第十次党代会和市第五次党代会作出的战略部署，加快实施创新驱动发展战略和供给侧结构性改革，全面组织实施“七大攻坚战”，努力把南平建设成为生态文明的先行区域、绿色产业的新兴基地、幸福宜居的美好家园。

根据“十三五”规划和市第五次党代会精神，今后五年全市经济社会发展的主要预期目标是：

——综合经济实力大幅提高。到2021年，全市生产总值突破2100亿元，力争经济发展增速达到或超过全省平均水平，国定、省定贫困人口如期全面脱贫、5个扶贫开发重点县如期脱帽，与全国全省同步全面建成小康社会。

——城乡环境更加宜业宜居。实施武夷新区、延平城区和县域中心城市、美丽乡村建设“千亿投资计划”，南平行政中心顺利搬迁武夷新区，延平城区城市功能不断完善，县域中心城市承载力不断增强，美丽乡村展现新貌。

——发展内生动力日益增强。全力推动主导产业高端化、新兴产业规模化、特色产业集群化，高新技术产业比重明显提升，现代服务业发展明显提速，现代农业发展明显提效，培育一批千亿产业和百亿企业集团，构建竞争力强的现代绿色产业体系。

——生态文明建设成效显现。国家生态文明试验区改革试点工作走在全国全省前列，生态环境质量保持全国全省领先，森林覆盖率达77.5%以上，城市空气优良天数比例达95%以上，主要流域水质功能优良比例达100%，生态安全保障体系基本建成。

——人民生活水平全面改善。把发展的成果更多惠及人民群众，实现民生投入同步增长、居民收入同步提高、社会事业同步推进，社会保障体系更加完善，基本公共服务均等化水平稳步提高，社会更加和谐稳定，人民群众幸福指数全面提升。

为了实现上述目标，今后五年发展的总体思路是：突出一个主题，即坚持发展第一要务，进一步解放思想，破除一切束缚发展的羁绊，加快实施绿色发展战略，以更高站位、更新理念谋划发展，突破大产业大项目，突出大招商招大商，推动大建设大发展。抓住两个重点，即一手抓城乡建设，一手抓产业发展。城市是产业的载体，产业是城市的支撑。没有好的城市就没有好的产业，没有好的产业就支撑不起好的城市。要抓好城市建设，筑巢引凤推动产业发展；要加快产业发展，增强对城市的支撑，实现以城促产、以产兴城、产城融合。积极推动美丽乡村建设与现代农业、乡村旅游发展的有机融合，通过建设美丽乡村推动现代农业和乡村旅游发展，通过发展现代农业和乡村旅游带动美丽乡村建设，让乡村更美、农业更强、农民更富。强化三个保障，即政策、机制和人才保障。要拓宽视野、打破常规，制定更具含金量、更富前瞻性的政策措施，以政策突破释放发展潜能；转变观念、强化运作，创新推动发展的体制机制，以体制机制创新激活发展动能；重视人才、重用人才，打造更加优惠的引才育才“洼地”，以人才集聚支撑发展提速。营造四个环境，即营造公平高效、充满活力的市场环境，山清水秀、宜业宜居的生态环境，互利共赢、共同发展的营商环境，风清气正、和谐稳定的社会环境，把南平打造成为一流的投资热土和创业福地。

2017年是新一届政府履行职责的开局之年，是实施创新驱动发展战略和供给侧结构性改革的深化之年。经济社会发展的主要预期目标是：全市生产总值增长8.5%，一般公共预算总收入和地方一般公共预算收入均增长5%，固定资产投资增长15%左右，外贸出口增长3%，实际利用外资增长10%，社会消费品零售总额增长10.5%，居民消费价格涨幅3%左右，城镇登记失业率控制在4.2%以内，人口自然增长率控制在9.5‰以内，城镇居民人均可支配收入增长8.5%，农村居民人均可支配收入增长9%，完成省下达的年度节能减排降碳任务。

今年重点要抓好以下八个方面工作：

（一）统筹加快城乡发展，全力建设美丽南平

全面实施新一轮城市总体规划，完善城市控制性详细规划、专项规划和城市设计。推进“多规合一”，强化规划管控，合理划分国土开发空间，留足公共空间和发展备用空间。创新城市综合管理方式，推动城市卫生保洁、市政园林和设施维护等社会化管理，打造山水相融、城景相依的绿色城乡、水美城乡和海绵城市。坚持分类调控、因地施策，满足群众住房刚性需求，促进房地产市场健康发展。以“智慧南平”为抓手，积极推进三网融合，加快城乡信息化建设。

全力建设武夷新区。围绕行政中心搬迁的目标，集聚要素、强力推动武夷新区开发建设。继续全面优化提升新区的城市规划设计、景观风貌、建筑风格，梳理明晰童游、将口、兴田三大片区功能定位，在城市建设上注重与建阳区统筹规划，实现同城发展；在产业发展上注重与建阳区、武夷山市相互衔接，推动产城互动。全面加快南林核心区“一纵三横”路网、绕城高速、云谷小区、市民服务中心、综合医院、文化艺术中心、体育中心、党校、宾馆、地下综合管廊、水资源配置和供电工程等重点项目建设，引导发展要素向核心区集中集聚。积极推动兴田片区万达文旅综合体、航天主题博览园、中国人寿国寿嘉园、森林生态旅游小镇、海峡医养产业园等项目开工建设，加快打造兴田体育、文旅、健康养生基地。积极推进将口片区武夷山东站站前广场的项目建设，继续加快303快速通道、轨道交通项目建设，结合崇阳溪景观带建设，打造以马拉松赛道、自行车道、步行道为主体，集体育、文化、旅游为一体的崇阳溪特色产业走廊。积极推进武夷高新技术产业园区建设，尽快完善服务配套设施，引进培育一批高新技术企业，加快软件园、铝轻量化专用车、大容量动力锂电池、海源新材料等项目建设。

加快建设延平城区。坚持新老城互动、山水城融合和产城融合的理念，老城区加快“两横四纵”主街道立面改造和一批市政道路、桥梁、隧道、公共停车场、农超市场、城市公园等补短板项目建设，让城市更加宜居。新城区重点推进高铁北站片区、徐洋溪片区、延平新城港区以及西站片区城市综合开发，加快产业园区建设，引进一批“五南”企业的中下游产业，大力发展新兴产业，配套完善教育、医疗、文体等公共服务设施。加快“一湖三江六岸”景观整治和九峰山、马头山森林公园以及316国道城市快速通道改造、闽江大桥八仙隧道连接线、九峰山慢道等项目建设，启动滨江东路、有轨电车、水上交通等项目前期，促进新老城区融通互动、产城融合。

推进建设荣华山组团。坚持“持续发展、统一规划、产城融合、创新运作”的原则，进一步理顺荣华山组团管理体制和建设机制，优化浦城县和荣华山组团产城一体化规划，通过公司化运作，提升融资运作能力，推进基础设施、市政设施和公共服务配套设施建设，促进荣华山组团与浦城县产城融合发展。

做精做美县域中心城市。将县域中心城市建设与中小流域治理、旅游景区建设等有机结合起来，策划实施一批市政基础设施和公共服务配套项目，补齐城市短板，完善城市功能，拉开城市框架，继续推进市政提升“六千工程”和“三边三节点”工程，加快宜居环境建设，做大做美县域中心城市。

建设特色小镇和美丽乡村。突出闽北特色风貌，加大对历史文化名镇、名村和古村落、古建筑的保护力度，深化“万人保洁”长效机制，加强高铁、高速公路及江河沿线的环境整治和景观提升，着力整治改善乡村人居环境，打造一批特色小镇、美丽乡村示范点，使美丽的南平大地显山露水、留住乡愁。

（二）强力推动项目建设，全面扩大有效投资

项目建设是扩大有效投资、增强发展后劲的重要抓手，必须以更大的气魄和力度来策划生成项目，推动项目落地建成。全年推进省市“五个一批”项目2150个、总投资7900亿元；实施在建行动计划重大项目173个，计划投资258亿元；实施省市重点项目381个，计划投资469亿元；实施20个工程包730个项目，计划投资81亿元。

全方位多层次谋划项目。建立市、县领导策划项目和专业机构策划项目的工作机制，提升项目策划的层次和水平。立足我市的资源禀赋，紧盯国家、省的政策导向和投资方向，着眼市场和民生需求，对标发达地区，按照“缺什么补什么”的原则，加快策划实施一批基础设施、城乡建设、产业发展、社会事业与民生保障项目。

大力度高效率推进项目落地建设。一要抓好项目前期。加大项目前期投入，鼓励服务外包，高效率、高质量地做好项目前期。二要抓好项目征迁。组织精兵强将，以会战的方式进行攻坚，强力推进征地拆迁，切实保障项目建设用地。三要抓好项目融资。积极争取中央、省预算内资金和国家专项建设基金，通过发行债券、政府购买服务、融资租赁、PPP等方式，加大项目融资力度，满足项目建设的资金需求。四要抓好项目管理。更加注重项目规范化操作、标准化建设、精细化管理，确保工程质量和安全，努力打造精品工程。要加强“五个一批”项目滚动管理，完善“五个一”项目建设机制，推行“一线工作法”，每季度集中开竣工一批项目，实行“一季一公布、一季一督查”，全力加快项目建设步伐。全年力争宁德至南平铁路、吉武温铁路、武夷山机场迁建、武沙高速、鹰厦铁路北段扩能改造等一批项目前期工作取得明显进展，宁武高速武夷山北城互通、太阳高新材料等项目开工建设，衢宁铁路、顺邵高速、南纺高新材料产业园等项目加快建设，南平联络线、南三龙铁路、华电邵武火电厂扩建、太阳电缆无氧铜杆及铜线一期工程等项目竣工投产。

（三）注重从供给侧发力，加快产业转型升级

以创新驱动为重要动力，以供给侧改革为主攻方向，全力抓龙头、铸链条、建集群、促转型，加快打造南平产业升级版。

加快培育壮大优势产业。坚持做优增量与盘活存量并重，设立百亿产业发展基金，推动产业结构优化升级。做优增量，就是要加快实施新兴产业倍增计划，集中力量在产业政策、园区建设上给予重点支持，加快推动新一代电子信息、新材料、新能源、节能环保、生物医药等战略性新兴产业发展。盘活存量，就是要用新技术新业态全面改造提升传统产业，推动食品加工、竹加工、机电制造、新型轻纺等产业转型升级，鼓励现有龙头企业增资扩产，延伸上下游产业链，打造产业集群。加快实施100个工业投资（技改）项目、30个智能制造和两化融合项目，推动传统产业向价值链中高端延伸。下大功夫发展实体经济，优化民营经济发展环境，进一步研究出台帮扶企业降成本、拓市场系列政策措施，大力支持企业在“新三板”挂牌和创业板、主板上市，加快推进企业兼并重组，扶持企业做大做强。着力实施创新驱动发展战略，坚持自主创新与借智借力有机结合，设立创业创新投资基金，支持引导科研机构、高等院校与企业、行业协会设立研发中心、科技创新基地、技术服务平台和众创空间，提升企业创新能力。支持圣农集团肉鸡产业化发展，加快培育茶、酒、肉制品、乳制品、食用菌等特色产业，提升精深加工水平，打响“武夷食品”品牌。

加快发展现代服务业。坚持市场需求导向，加快培育生活性服务业和生产性服务业，推动现代服务业发展提速、比重提高、效益提升。围绕建设“国际养生旅游目的地”和“全域旅游目的地”，依托武夷山“双世遗”品牌优势，创新旅游宣传营销模式，打响“清新福建、快乐武夷”品牌，深化旅游管理体制改革，组建旅游发展委员会和旅游集团，打造“大武夷”旅游经济圈，加快大型旅游综合体建设，推动旅游与农业、工业、文化、体育、康养等产业深度融合，重点发展旅游休闲、体育健康、医疗养生等产业，丰富旅游新业态，实现从单一的观光游向休闲游、养生游转变，旅游观光地向旅游目的地转变，力争全年旅游接待总人数、旅游总收入分别增长15%、18%。加快建设大型商贸市场，促进商贸服务业提档升级。加快启动建设武夷新区、延平新城综合物流园区和光泽冷链物流园区，完善现代物流服务体系；建设“茶叶交易中心”，将其打造成为具备现货交易、线上线下交易、仓储物流、标准检测、支付结算和金融服务等功能的交易平台。加快规划建设武夷新区金融后台集聚区，支持更多银行、证券、保险、信托、金融租赁公司、基金公司等金融机构和类金融机构来我市发展。加快发展总部经济，积极对接、洽谈和引进一批企业总部或区域事业部。加快推进文化创意、会展培训、电子商务和家政服务等现代服务业发展。

加快建设特色现代农业。深入推进农业供给侧结构性改革，坚持现代化、规模化、产业化和信息化的“大农业”发展理念，大力发展品牌农业、智慧农业、生态农业和设施农业，推动农产品生产与加工、流通、服务、旅游有机结合，促进一产接二连三融合发展。大力发展品牌农业，按照“三品一标”要求，进一步推进农业品种创新、品质提升、品牌打造。大力发展智慧农业，培育一批农业“五新”示范项目，推进“互联网+现代农业”，加快信息技术在农业生产、流通、服务、管理全领域应用。大力发展生态农业，优化农业发展布局，减少化肥农药使用，强化农业废弃物资源化利用，加强农产品质量安全追溯体系建设，推进农业可持续发展。大力发展设施农业，推进高标准基本农田建设，加快“一区二园八基地”建设，打造一批产业集聚、生产标准、服务配套的现代农业产业园。着力引进一批农业龙头企业，培育一批新型农业经营主体和服务主体。扎实推进第三次全国农业普查。

（四）坚持大招商招好商，提升对外开放水平

招商引资是增强内生动力、发展动能的重要途径，要进一步加大招商引资力度，力争引进投资超亿元合同项目260个、投资超2000万元新开工项目280个以上。

增强招商引资实效。一要突出精准招商。制定更加精准的招商扶持政策，进一步拓宽招商领域，将招商的重点从传统产业延伸至高新技术产业，从产业领域延伸至社会事业、民生保障等领域。坚持引龙头、铸链条，紧盯世界、中国500强企业、知名公司、行业龙头等重点对象，引进一批“补链”“强链”“扩链”的大项目。二要创新招商方式。推行产业链招商、互联网招商、中介招商以及龙头企业以商引商等多种招商方式，充分运用好各类重大展会，创新“11·16”茶博会办会模式。发挥异地商会作用，深入实施“回归工程”，促进企业回乡、人才回归、资金回流。在招商引资过程中，一方面要防止“饥不择食”，坚守生态环保底线；另一方面要提倡“饿虎扑食”，对引进的大项目好项目要咬住不放，成立专门跟踪服务工作组，实行领导挂钩负责，全力推动项目落地。三要搭建招商平台。把产业园区作为招商项目落地的主要平台，加快推动园区生产专业化、生活社区化、环境生态化，尽快完善园区的市政和公共服务配套设施，提升招商引资的吸引力和落地率。

提升对外开放水平。立足“万里茶道”，融入“一带一路”，积极对接可复制的自贸区政策。以国台办授予我市“朱子故里海峡两岸交流基地”为契机，进一步加强对台文化交流和对台农业、旅游等产业合作。深化跨区域协作、山海协作，发挥南平旅港联谊会、澳门南平联谊会的窗口平台作用，增强经贸交流合作实效。建立“大通关”协调机制，发展外贸新业态，扩大外贸出口。加大武夷山机场航班航线培育力度，推动航空口岸扩大开放的申报工作。

（五）凝聚合力精准施策，深入推进脱贫攻坚

更加广泛凝聚合力。落实攻坚责任，统筹扶贫资源，创新体制机制，强化支撑保障，建立健全各级领导干部挂钩帮扶贫困村、贫困户机制，深化部门挂钩，实现结对帮扶全覆盖。广泛动员全社会力量参与扶贫，加快构建政府、行业、社会协同推进的大扶贫格局，确保全年脱贫3万人以上。

更加精准施策帮扶。强化产业扶贫、就业扶贫，鼓励龙头企业、农村合作组织与贫困户建立利益联动机制。依托县城、集镇、工业园区、中心村安置搬迁群众，完成造福工程搬迁1万人以上，确保搬得出、稳得住、能致富。实施健康扶贫工程，深化医疗救助，探索开展可持续的商业保险，降低因病致贫返贫风险。实施教育扶贫，阻断贫困代际传递。推进农村低保制度与扶贫开发政策有效衔接，加大贫困人口基本生活、养老等保障力度。强化脱贫攻坚工作督查，严格扶贫开发项目资金监管，建立健全稳定脱贫长效机制，完善贫困户动态管理制度。

（六）加强生态文明建设，持续保护绿水青山

牢固树立“绿水青山就是金山银山”的理念，加快建设全国绿色发展示范区，让美丽南平的山更青、水更绿、空气更清新。

主动融入生态文明试验区建设。加快实施国家生态文明试验区行动方案，继续推进武夷山国家公园体制、生态系统价值核算、重点生态区位商品林赎买和党政领导干部自然资源资产离任审计等试点工作，加快分步实施115项、总投资793亿元的生态文明试验区重大项目，形成一批可复制、可推广的试验成果。建立生态文明建设考评体系，健全环境资源保护管理制度，加强生态红线管控，推行环保责任险制度，推动生态损害赔偿试点。争取发行绿色专项债券，逐步推动用能权、碳排放权和排污权交易。

加大生态环境保护力度。推行第三方治理、合同环境服务、区域综合服务等市场化环境治理和生态保护模式，加快建设生态环境治理保护一体化环保园。大力发展循环经济，促进资源集约和节约利用。积极申报国家山水林田湖生态保护修复工程项目。切实落实水污染防治行动计划，实施“河长制”，构建责任明确、协调有序、监管严格、保护有力的河湖管理保护机制，加强闽江流域水环境和小流域、农村水环境综合整治，强力推进畜禽养殖污染治理，打造“清新水域”。切实落实大气污染防治行动计划，推进区域联防联控和预警预报，加强重点行业企业大气污染物综合治理，全面淘汰“黄标车”，打造“清净蓝天”。切实落实土壤污染防治行动计划，开展土壤污染状况详查，积极推行垃圾分类处理，加快城乡生活垃圾、建筑垃圾综合整治，推行固废、危废、医废、餐厨废弃物等垃圾无害化处理，加大茶山整治和重点区域水土流失治理力度，打造“清洁土壤”。

（七）深化体制机制创新，不断激活发展动力

按照统筹推进、重点突破的要求，纵深推进体制机制改革创新，向改革要动力、要活力、要效益。

深化投融资体制创新。把握金融政策“窗口期”，建立全市投融资一盘棋机制，创新融资方式，拓宽融资渠道。运用产业基金，以股权方式投入，支持处于不同发展阶段的企业逐步壮大，实现财政资金保值增值和循环使用。加大公司化融资运作力度，积极争取地方政府债券，做好政府债务置换，合理控制政府新增债务规模，降低融资成本，做好还贷源的准备。扎实做好金融风险防范工作，全力化解不良资产，力争全市不良贷款率控制在3%以内。

推进重点领域改革。分类推进市属国有企业改革，健全现代企业制度，加快国企整合重组，组建金控公司、资产管理公司和国有资本投资运营公司；突出国有企业主业定位，实施经营性项目、非经营性项目分类考核，采取有效资产注入，提高国有企业信用等级，使其做强做大，充分发挥国有企业在建设发展中“当先锋、打头阵”的作用。深入推进“放管服”改革，借鉴先进地区和自贸区项目审批做法，建立权责清单动态调整和规范运行机制，推行“一照一码”“两证整合”“五证合一”，加强网上审批，规范中介服务。推进公安工作改革，深化供销合作社综合改革。稳妥推进农村土地“三权分置”，基本完成农村土地承包经营权确权登记颁证。

完善人才保障机制。制定出台更加优惠、更有针对性的政策措施，设立人才发展基金，在住房保障、薪金收入、配偶随迁、子女就学、创业融资、产权保护、收益分配等方面，阶梯式地提高人才待遇，更加精准和灵活地引进高端人才，形成育才聚才用才的政策洼地，为更多优秀人才搭建创业发展的平台。

（八）加快发展社会事业，全面增进民生福祉

坚持以人为本，把更多新增财力用于民生事业，认真办好为民办实事项目，扩大公共服务产品有效供给，使人民群众有更多的获得感。

优先发展教育事业。实施教育强市战略，优先保障教育投入，巩固义务教育基本均衡县国检达标成果，更高水平统筹城乡义务教育均衡发展。实施学前教育三年行动计划，推进中小学扩容提质和优化布局，积极创建优质高中，提高基础教育普惠水平。创新职业教育发展方式，提高职业教育与产业发展契合度。支持武夷学院等高等院校转型发展。发展特殊教育、终身教育和老年教育。实施名师名校培养工程，加强教师队伍建设。

加快提升医疗水平。持续深化医药卫生体制改革，加快建立和完善分级诊疗制度，鼓励社会资本参与办医，加快发展高端医疗和专科医院，切实改善群众医疗条件。开展名医评选活动，加强全科医生和乡村医生队伍建设，提高城乡医疗服务水平。健全基本医疗保障制度，推进城乡居民医疗保险一体化。落实全面“两孩”政策，推动计生工作由管理向服务转变。

推进文化体育繁荣发展。加强文化资源保护利用，积极引导公共文化服务向基层延伸，突出打响朱子文化、红色文化等品牌，加快朱子学院、考亭朱子文化园、五夫朱子文化生态园、延平书院等项目建设，开发建盏、建本等特色文化产品，加快发展新闻出版广播影视事业，促进文化繁荣。加快发展体育事业，推动群众体育和竞技体育全面发展，着手申办第十七届省运会。积极创造条件争创省级文明城市。

创新社会治理。深入实施“七五”普法。深化“平安南平”创建，突出预警防范，加强反恐怖工作，构建全方位、立体化的社会治安防控体系，加强和改善信访工作，有效化解矛盾纠纷，促进社会和谐稳定。深化安全生产责任体系标准化建设，加强安全生产大排查，推进重点行业、重点领域安全生产整治，强化食品药品安全监管，深化“餐桌污染”治理。加强救灾物资储备库建设，提升防灾减灾和应急处置能力。

强化社会保障。健全完善社会保险、社会福利、社会救助、社会慈善等多层次社会保障体系，做好妇女、儿童、老人和残疾人关爱服务工作，新扩建3个县级社会福利中心，建设一批乡村敬老院、幸福院和城市社区老年照料中心。加强社区、农村服务管理，适当提高社区工作者和村居干部待遇。加大棚户区改造力度，全面完成省下达的保障性安居工程任务。实施更加积极的就业政策，推动更高质量就业。

与此同时，要抓好新一轮全省“双拥模范城（县）”创建工作，支持驻军部队和武警部队建设，加强国防建设和人民防空建设。加强与各民主党派、工商联、无党派人士的合作共事和民主协商，支持工会、共青团、妇联等群团组织开展工作，关心离退休老干部，积极做好民族宗教、外事侨务、档案方志、气象防震、老区库区等各项工作。

三、加强政府自身建设

（一）进一步解放思想，勇于创新运作。要不断加强学习，勇于突破与发展不相适应的思想观念、思维方式，敢于运作、善于运作。勇于结合本地实际，大胆创新体制机制，走别人没有走过的路，走以前没有走过的路，走出去、请进来，借鉴学习先进地区的好经验好做法，着力提升干部在城市规划建设管理、项目谋划策划、推动产业发展、招商引资等方面的能力，更好推动发展提速进位。

（二）进一步担当尽责，提升效能效率。要认真落实中共南平市委《关于加强市县乡领导班子思想政治建设的实施意见》和《关于强化担当尽责推动工作落实的若干意见》，建立健全正向激励机制，落实容错会审制，鼓励干部敢担当、敢负责、有作为，形成干事创业的良好氛围。要建立目标倒逼工作和问责工作机制，倡导“马上就办”，加大督查力度，层层传导压力，对看准议定要办的事，就要雷厉风行、强力推进、一抓到底，确保各项工作部署落地生根。要深化效能建设，着力破解“不冷不热、不急不忙、不疼不痒”等不作为、不担当的问题，提高办事效率。

（三）进一步依法行政，建设法治政府。要深入贯彻依法治国基本方略，把政府工作全面纳入法治轨道，不断提高政府工作的法治化水平。加强政府诚信建设，提高政府公信力。自觉接受人大、政协监督，认真办好人大代表议案、建议和政协委员提案。主动接受司法、舆论和群众监督，加强行政监察和审计监督。

（四）进一步优化环境，提升服务水平。要建立健全挂点帮扶企业、项目机制，开展“百名局长帮扶百家重点企业”“千名干部服务千个重点项目”活动，在主动、精准、实效上下功夫，增强主动服务、超前服务、创新服务的能力，坦荡真诚地同企业家接触交往，让政商关系在“清”的前提下“亲”起来。

（五）进一步正风肃纪，坚持廉洁从政。全面推进从严治党，严格执行党内政治生活若干准则、廉洁自律准则和纪律处分条例、问责条例、党内监督条例，持之以恒落实中央八项规定精神，深入反对“四风”，积极践行“三严三实”。严格落实“一岗双责”，强化对权力运行的监督和制约，全面推进党风廉政建设和反腐败工作，确保权力在阳光下运行，树立政府清正廉洁的良好形象。

各位代表，全面建成小康社会、建设全国绿色发展示范区的目标催人奋进。让我们紧密团结在以习近平同志为核心的党中央周围，在省委、省政府和市委的坚强领导下，紧紧依靠全市人民，围绕“机制活、产业优、百姓富、生态美”的目标，坚定信心，锐意进取，铆足干劲，撸起袖子加油干，为再上新台阶、建设新南平作出新的更大贡献！
